# CD34^−^ human placenta-derived mesenchymal stem cells protect against heat stroke mortality in rats

**DOI:** 10.18632/oncotarget.23324

**Published:** 2017-12-15

**Authors:** Willie Lin, Yogi Chang-Yo Hsuan, Yu-Chin Su, Cheng-Hsien Lin, Mao-Tsun Lin, Zi-Hao Chen, Ching-Ping Chang, Kao-Chang Lin

**Affiliations:** ^1^ Meridigen Biotech Co., Ltd., Taipei, Taiwan; ^2^ Department of Medical Research, Chi Mei Medical Center, Tainan, Taiwan; ^3^ Institute of Molecular Medicine, College of Medicine, National Cheng Kung University, Tainan, Taiwan; ^4^ Department of Biotechnology, Southern Taiwan University of Science and Technology, Tainan, Taiwan; ^5^ The Ph.D. Program for Neural Regenerative Medicine, Taipei Medical University, Taipei, Taiwan; ^6^ Department of Neurology, Chi Mei Medical Center, Tainan, Taiwan

**Keywords:** heat stroke, placenta, mesenchymal stem cells, ischemia, inflammation

## Abstract

CD34 is a transmembrane phosphoglycoprotein used to selectively enrich bone marrow in hematopoietic stem cells for transplantation. Treating rats with CD34^+^ cells derived from human umbilical cord blood before or after heat stroke has been shown to promote survival. We investigated whether CD34^–^ human placenta-derived stem cells (PDMSCs) could improve survival following heat stroke in rats. Rats were subjected to heat stress (42°C for 98 min) to induce heat stroke. Intravenous administration of PDMSCs 1 day before or immediately after the onset of heat stroke improved survival by 60% and 20%, respectively. Pre-treatment with CD34^−^ PDMSCs protected against heat stroke injury more effectively than that treatment after injury. PDMSCs treatment attenuated cerebrovascular dysfunction, the inflammatory response, and lipid peroxidation. These data suggest human PDMSCs protect against heat stroke injury in rats. Moreover, these effects do not require the presence of CD34^+^ cells.

## INTRODUCTION

Cardiovascular disease is the primary cause of death in the United States [1]. An inverse correlation between cardiovascular disease and the number and function of circulating angiogenic cells (CACs) was reported previously [2]. CACs are endothelial progenitor cells, which are categorized based on the expression of cell surface markers such as CD34. CD34^+^ cells have been evaluated for the treatment of ischemic conditions [3, 4]. Systemic administration of CD34^+^ cells derived from human umbilical cord blood (HUCB) was shown to reduce heat stroke-induced mortality in rats [5, 6].

CD34^−^ cells have also been investigated for the treatment of several cardiovascular diseases. For example, mononuclear cell (MNC) fractions isolated from HUCB that were depleted of CD34^+^ stem cells reduced ischemic brain injury in a rat model of stroke [7, 8]. Additionally, CD45^+^/CD34^−^/Lin^–^ stromal cells derived from bone marrow protected against ischemia and reperfusion injuries in rats [9]. Finally, circulating CD31^+^/CD34^−^ angiogenic cells improved hind limb ischemic injuries in mice [10].

The human placenta is a source of mesenchymal stem cells that have the ability to differentiate into cardiomyocytes, smooth muscle cells, osteoblasts, adipocytes, endodermic pancreatic islet cells, liver cells, ectodermic neurons, and astrocytes [11–13]. Flow cytometry analysis has indicated that human placenta-derived mesenchymal stem cells (PDMSCs) are CD34^−^ [14–16]. PDMSCs have been shown to promote healing in animal models of myocardial infarction, Parkinson's disease, diabetes mellitus, and spinal cord injury [14, 17–20].

In this study, we investigated whether CD34^−^ PDMSCs could improve survival in a rat model of heat stroke by altering the cerebrovascular, inflammatory, and lipid peroxidation responses.

## RESULTS

### PDMSCs treatment improves survival in a rat model of acute heat stroke

**Figure 1:**
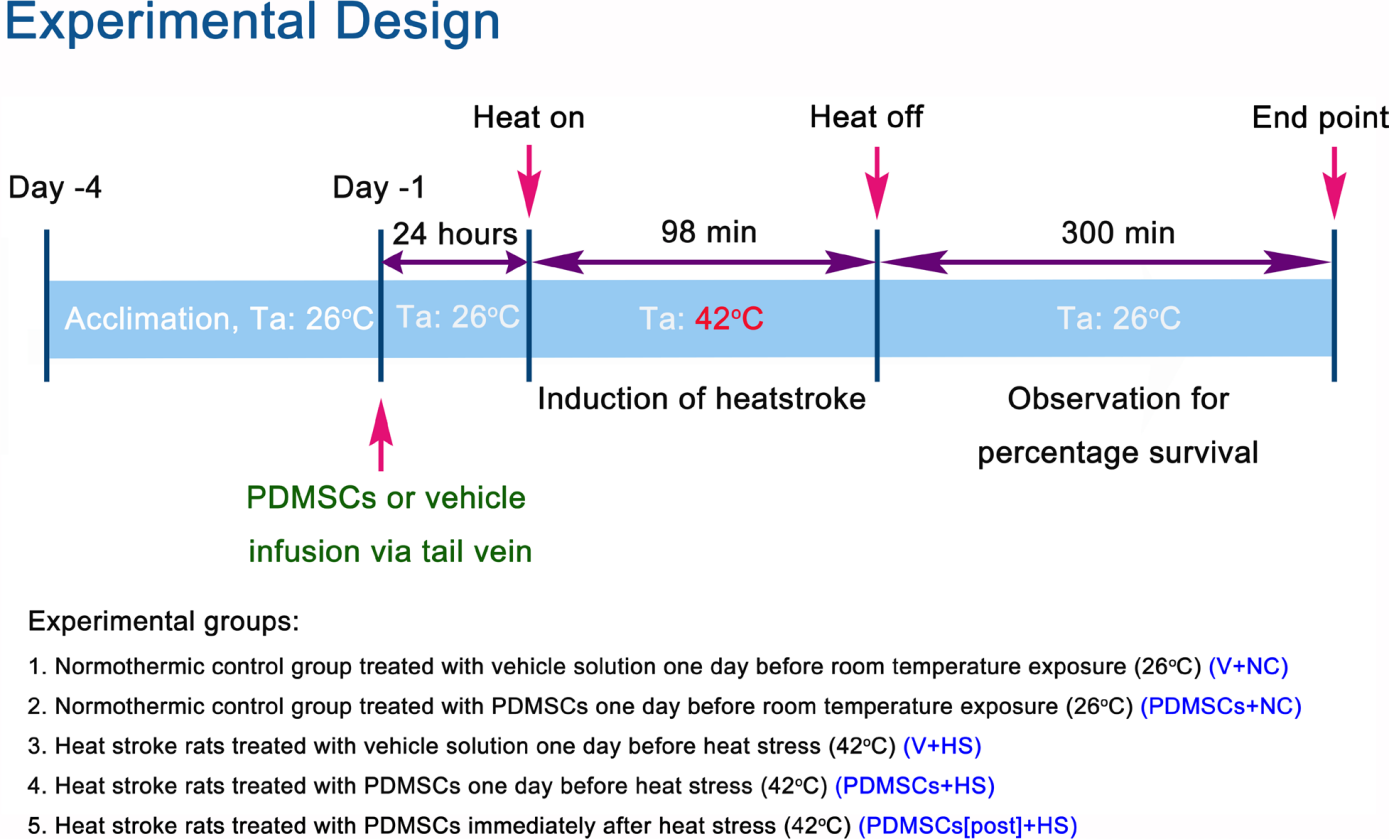
Experimental design and definitions of groups

**Figure 2:**
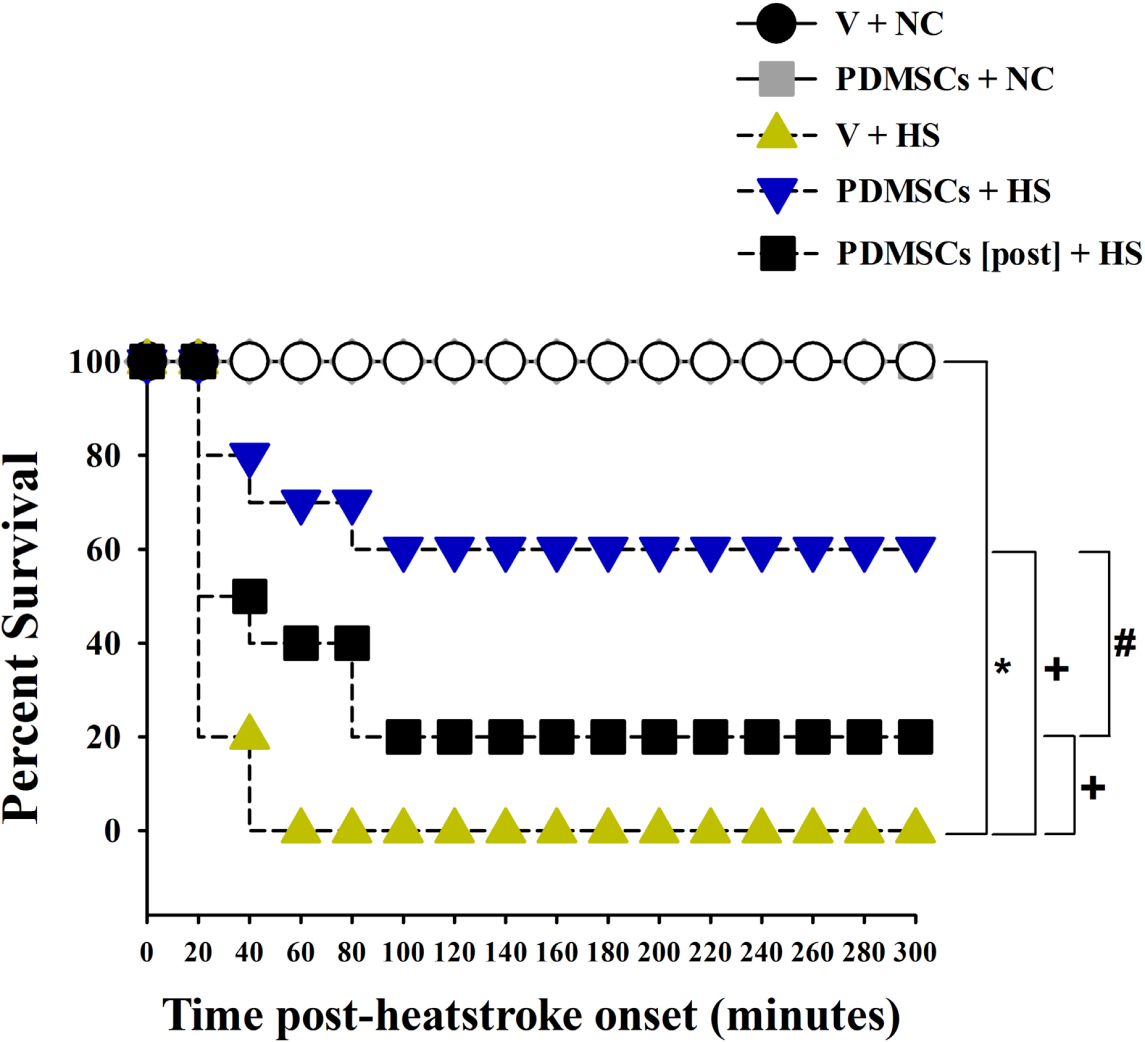
Kaplan-Meier analysis followed by log-rank tests were performed to determine percent survival in each group (*n* = 10) ^*^p < 0.05 vs. the NC group, ^+^*p* < 0.01 vs. the V+HS group. ^#^*p* < 0.05 vs. the PDMSCs+HS group.

The experimental design is illustrated in Figure 1. Following heat stress at 42°C for 98 min, rats were maintained at room temperature (26°C) and survival monitored over 300 min. Survival was lower among rats treated with vehicle solution 1 day before heat stress (V+HS) compared to normothermic control rats treated with vehicle solution 1 day before room temperature exposure (0% vs. 100%, respectively) (Figure 2). In contrast, survival was higher among heat stroke rats treated with PDMSCs 1 day before heat stress (PDMSCs+HS), and rats treated with PDMSCs immediately after heat stress (PDMSCs[post]+HS) compared to the V+HS group (60% and 20%, respectively, compared to 0%) (Figure 2). The protective effects of pre-treatment with PDMSCs were superior to those of post-treatment (Figure 2).

### PDMSCs treatment reduces hyperthermia, hypotension, and cerebral ischemia after heat stress

**Figure 3:**
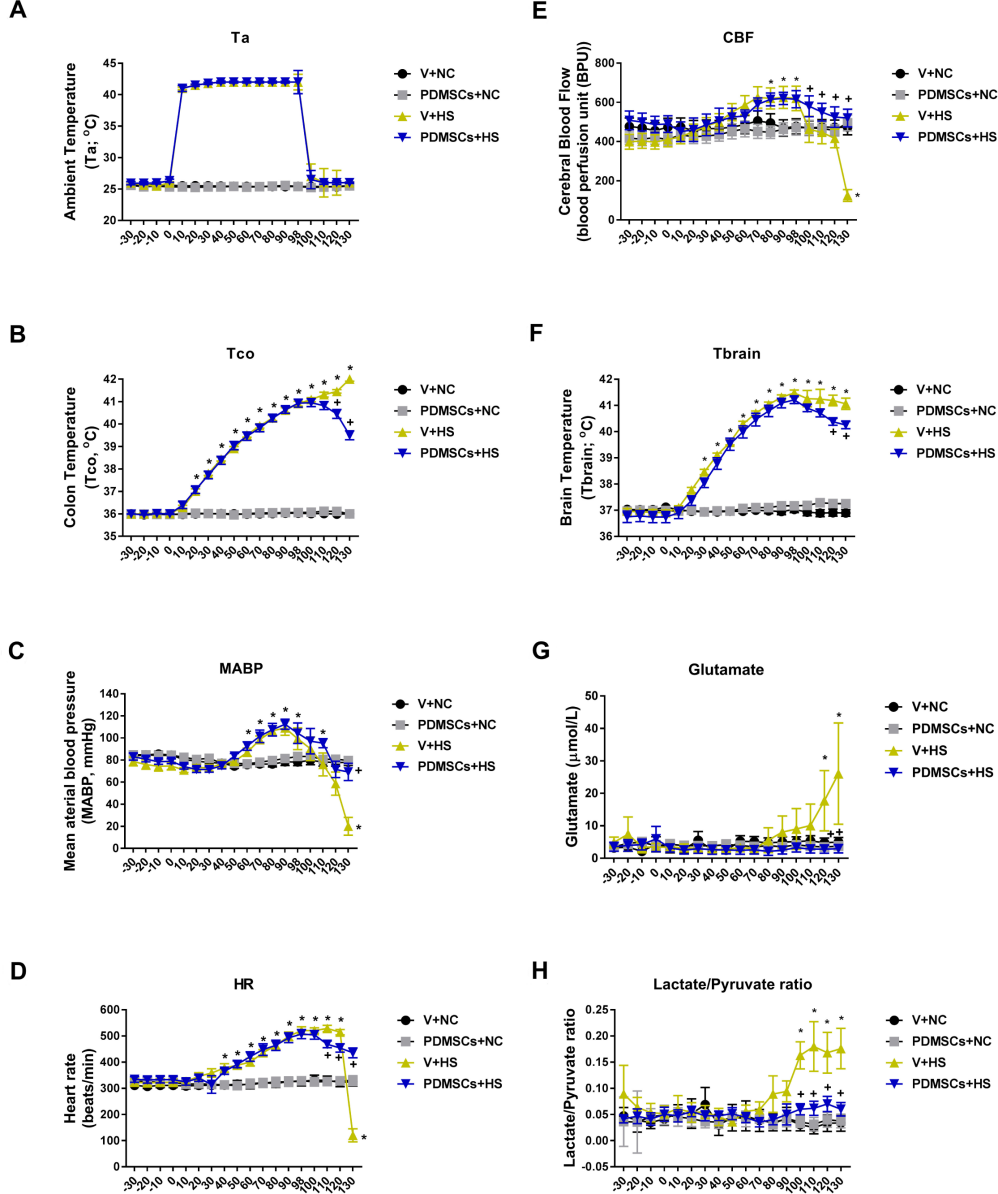
Functional recovery after PDMSCs treatment in a rat model of acute heat stroke PDMSCs were administrated intravenously 1 day before heat stress and the physiological response analyzed in each group (*n* = 10). Physiological functions include: (**A**) ambient temperature, Ta; (**B**) colon temperature, Tco; (**C**) mean arterial blood pressure, MABP; (**D**) heart rate, HR; (**E**) cerebral blood flow, CBF; (**F**) brain temperature, Tbrain; (**G**) brain levels of glutamate; and (**H**) brain levels of lactate/pyruvate ratio. *p* < 0.05 for the V+HS vs. V+NC group; ^+^*p* < 0.05 for the PDMSCs+HS vs. V+HS group. Data are presented as the mean ± S.D.

The V+HS group had higher body core temperatures 30 min following heat stress compared to the V+NC or PDMSCs+NC groups (41–41.5°C vs. 36–37°C, respectively) (Figure 3A and 3B). The V+HS group also had higher levels of several indicators of cerebral ischemia compared to the V+NC group (glutamate: 26 ± 15 μmol/L vs. 5 ± 1 μmole/L and lactate/pyruvate: 0.18 ± 0.04 μmol/L vs. 0.03 ± 0.02 μmol/L, respectively) (Figure 3G and 3H). In contrast, the V+HS group had a lower mean arterial pressure (MAP) (20 ± 5 mmHg vs. 78 ± 3 mmHg, respectively), heart rate (HR; 120 beats/min vs. 468 ± 52 beats/min, respectively), and cerebral blood flow (CBF; 200 ± 24 BPU vs. 400 ± 39 BPU, respectively) compared to the V+NC group (Figure 3C, 3D and 3E). PDSMC treatment reduced hyperthermia (Figure 3B and 3F), cerebral ischemia, hypotension, and bradycardia caused by heat stroke (Figure 3).

### PDMSCs treatment attenuates the inflammatory response and lipid peroxidation after heat stress

**Figure 4:**
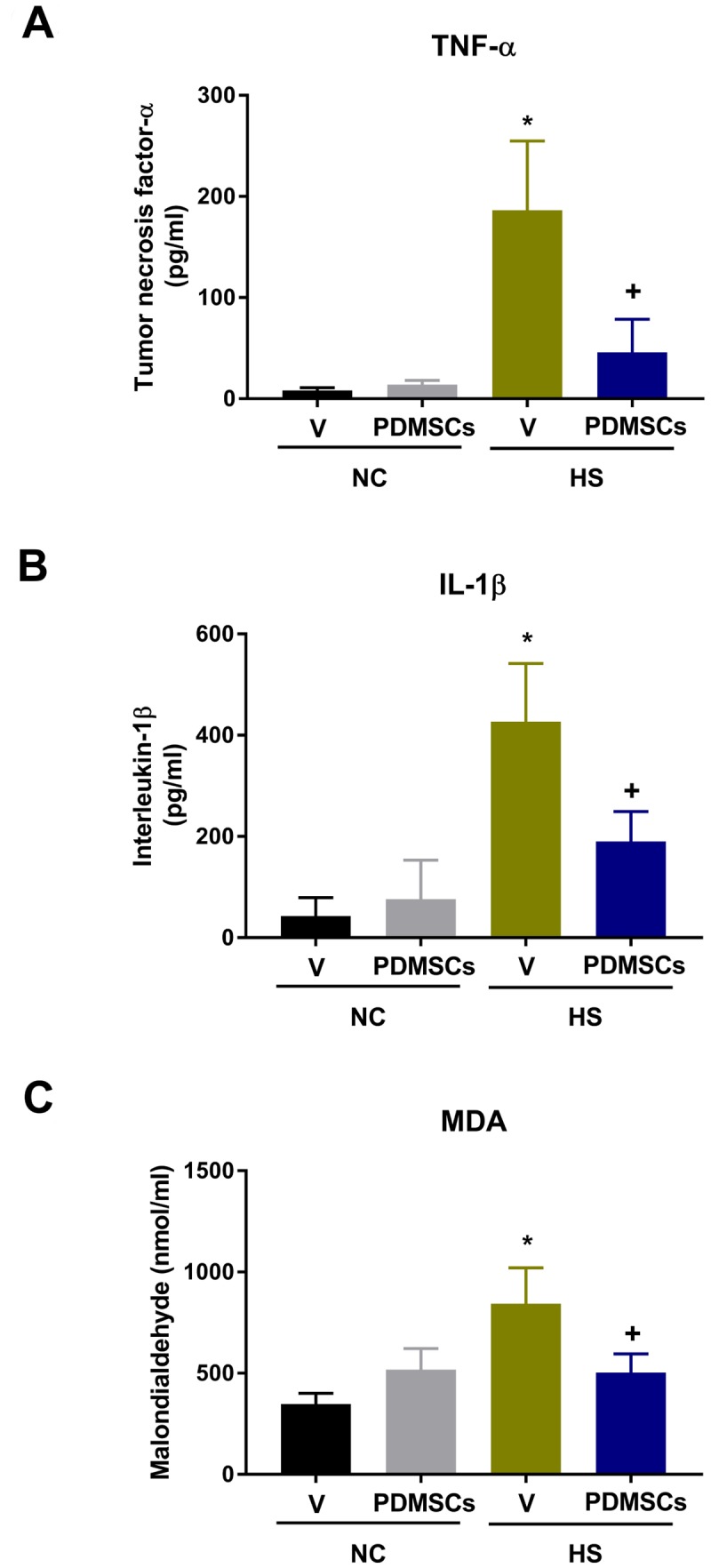
Analysis of the levels of (**A**) tumor necrosis factor-a (TNF-a), (**B**) interleukin-1b (IL-1b), and (**C**) malondialdehyde (MDA) following treatment with PDMSCs. PDMSCs were administered intravenously 1 day before heat stress, and the effects on serum TNF-α, IL-1β, and MDA analyzed in each group (*n* = 10). Data are presented as the mean ± S.D. ^*^p < 0.05 for the V+HS vs. V+NC group; ^+^*p* < 0.05 for the PDMSCs+HS vs. V+HS group.

The V+HS group had higher serum tumor necrosis factor-α (TNF-α, Figure 4A), interleukin-1β (IL-1β, Figure 4B), and malondialdehyde (MDA, Figure 4C) levels compared to the V+NC group 30 min after heat stress (193 ± 25 pg/mL vs. 8 ± 2 pg/mL, 427 ± 43 pg/mL vs. 43 ± 14 pg/mL, and 843 ± 67 nmol/mL vs. 518 ± 40 nmol/L, respectively) (Figure 4). Thus, PDMSCs treatment attenuates the inflammatory response and lipid peroxidation following heat stress.

## DISCUSSION

Environmental heat stress causes subcutaneous vasodilation, increased metabolism, splanchnic vasocontraction, hypotension, intracranial hypertension, and cerebral ischemia in rodents [21]. We found that vehicle-treated rats displayed hyperthermia, hypotension, cerebral ischemia and oxidative stress, and activation of the inflammatory response following heat stroke resulting in mortality. Intravenous administration of PDMSCs 1 day before or immediately after the onset of heat stroke increased the percentage survival to 60% and 20%, respectively, compared to 0% in controls. The protective effects of pre-treatment with PDMSCs were superior to those of post-treatment. PDMSCs therapy may attenuate heat stroke-induced mortality by attenuating hyperthermia, hypotension, cerebral ischemia and oxidative injury, and the inflammatory response.

Heat stroke contributes considerably to morbidity and mortality in a high environmental temperature [21]. About 30% of heat stroke survivors after whole body cooling, the current therapy of choice for heat stroke, experience disability and neurological dysfunction [22–24]. Many studies have demonstrated the beneficial effects of MSCs such as trophic factor-mediated neuroprotection, enhanced angiogenesis and neurogenesis, and modulate neuroinflammation [25]. In order to control the occurrence of the heat stroke, the prevention strategy is better than the post- heat stroke cure. Indeed our present data show that PDMSCs treatment immediately after heat stroke, the beneficial effect was inferior to those of pretreatment. Therefore, our results strongly recommend that a prophylactic dose of PDMSCs can be considered for those who intend to work or exercise in a hot environment.

Our data differ from those of previous findings supported by Chen and colleagues [6, 26]. They found that systemic administration of CD34^+^ cells derived from HUCB also promoted survival in heat stroke rats by suppressing the inflammatory response. In contrast, our present data showed that systemic administration of CD34^−^ PDMSCs shared with the CD34^+^ HUCB the same protective effects. Both CD34^−^ PDMSCs and CD34^+^ HUCB equally attenuated heat stroke-induced overexpression of both IL-1β and TNF-α in rats. These pro-inflammatory cytokines promote pyrogenesis, systemic inflammation, increased vascular permeability, and hypotension in response to heat stroke [27]. Putting together, PDMSCs may promote survival in heat stroke by a protective cell population without the presence of CD34^+^ cells.

Our findings are supported by data from other disease models. For example, intravenous administration of either HUBC-derived MNCs, MNC fractions enriched for CD34^+^ stem/progenitor cells, or MNC fractions depleted of CD34^+^ stem/progenitor cells reduced neurological injury and lesion volume in ischemic rats following stroke [28]. MNCs had the greatest effects compared to the other fractions. Intravenous administration of bone marrow-derived CD45^+^/CD34^−^/lin^−^ stroma cells protected against ischemia/reperfusion heart injury in rats [9]. Treatment with CD34^−^/CD31^+^ CACs improved mouse hind limb ischemia to the same degree as CD34^+^ cells [10]. Both HUCB MNCs and PDMSCs may protect against injuries caused by heat stroke through a protective cell population that has not yet been identified.

Rats that were not pretreated with PDMSCs prior to heat stress demonstrated a suppressed contractile function associated with stroke volume, ejection fraction, cardiac output, stroke work, and systolic pressure resulting in arterial hypotension. Similar results were described previously [29]. PDMSCs therapy could improve heat stroke-induced hypotension by increasing cardiac mechanical efficiency and decreasing arterial elastance.

We implanted a microdialysis probe into the hypothalamus of the rat brain to evaluate the impact of ischemia on the cells. If oxygen is available, pyruvate enters the citric acid cycle, which is the dominant producer of energy (ATP). Under ischemic conditions (inadequate blood supply), there is a decrease in the supply of oxygen and glucose. Therefore, there is an increase in cellular uptake of glucose in order to generate ATP through anaerobic glycolysis, which leads to a decrease in dialysate glucose concentration. This results in increased lactate production in order to generate NAD^+^ for anaerobic glycolysis and an increase in the lactate-to-pyruvate ratio [30, 31]. Under ischemic conditions, glutamate is released from neurons and initiates a pathological influx of calcium leading to cell damage [32]. Calcium influx and glutamatergic excitotoxicity leads to structural alterations in the inner mitochondrial membrane. Disorganization of the electron transport chain increases radical oxygen species formation, which can lead to necrosis or apoptosis [32]. We found that increased cellular levels of ischemia (e.g., glutamate and lactate-to-pyruvate ratio) and lipid peroxidation markers (e.g., MDA) in response to heat stroke were attenuated by PDMSCs treatment.

The plasma levels of pro-inflammatory cytokines such as TNF-α and IL-1β are elevated following heat stroke [21, 33]. The increase in the levels of these cytokines may be correlated with heat stroke severity. We demonstrated that CD34^−^ PDMSCs have anti-inflammatory effects similar to those of HUCB-derived CD34^+^ cells in rats following heat stroke. Both cell populations could improve outcomes following heat stroke by preventing the overproduction of pro-inflammatory cytokines including IL-1β and TNF-α.

Severe heat stress causes subcutaneous vasodilation and ischemia in multiple vital organs including both the heart and brain. Once recruited to the site of cardiac and/or cerebral ischemia, PDMSCs improve outcomes following heat stroke by stimulating tissue revascularization and neuroregeneration through differentiation into vascular or neuronal cells, the release of paracrine factors (e.g. growth factors, interleukins, neurotrophic factors), and through interactions between MSCs and the host tissue, which promotes vascular neogenesis and neuroregeneration [34, 35].

## MATERIALS AND METHODS

### Animal experiments

A total of 90 male Sprague-Dawley rats (10 weeks old, 248–329 g) were housed individually and allowed to access to food and water *ad libitum*. Animals were labeled with identifiers on the tail with a non-toxic, temporary pen. All procedures were performed under urethane anesthesia. Every effort was made to minimize the number of animals and limit pain and suffering.

### Induction of heat stroke

All Sprague-Dawley rats were obtained from BioLASCO Taiwan Co., Ltd. (Taipei, Taiwan) to exclude the possibility of gender-related differences in heat tolerance. The rectal temperatures of the anesthetized rats were maintained at 36 ± 1°C during the procedure using a water-circulating folded heating pad. The right femoral artery and vein were cannulated for measurement of the MAP and HR, and for cell delivery. Heat stroke was induced by increasing the temperature of the folded heating pad from 36°C to 42°C for approximately 98 min (Figure 1) [36]. Animals were then allowed to recover at room temperature (26°C).

### PDMSCs isolation and culture

Human PDMSCs were obtained from Meridigen Biotech Co., Ltd (Taipei, Taiwan). All donor women were 20–45 years old, negative for syphilis, HIV, CMV, HBsAg, and HCV, and had no history of infectious diseases or complications during pregnancy. Donors provided written informed consent. Placental tissue was harvested under sterile conditions, rinsed with PBS, and cut into 1 × 1 × 1 mm^3^ pieces with scissors. Next, the specimens were digested with 100 mL of 2 mg/mL collagenase (NB6 GMP Grade, SERVA) in a 37°C incubator for 120 minutes. The digestion was terminated with the addition of culture media. Cell suspensions were centrifuged at 300 RCF for 5 minutes. Supernatants were aspirated and the cells resuspended in 35 mL of complete media. Cell solutions were then seeded into T175 flasks (Nunc MaxiSorp; Thermo Fisher Scientific, Rockford, IL, USA). The cells were then cultured in a humidified incubator with 5% CO_2_ at 37°C. After 3 days, the culture media was replaced and non-adherent cells removed. The media was exchanged every 2–3 days. PDMSCs were passaged once they reached 80–90% confluence. For long-term storage, they were resuspended in CryoStor^®^ CS10 and stored in a vapor phase liquid nitrogen tank.

### Identification of PDMSCs and analysis of differentiation capacity

**Figure 5:**
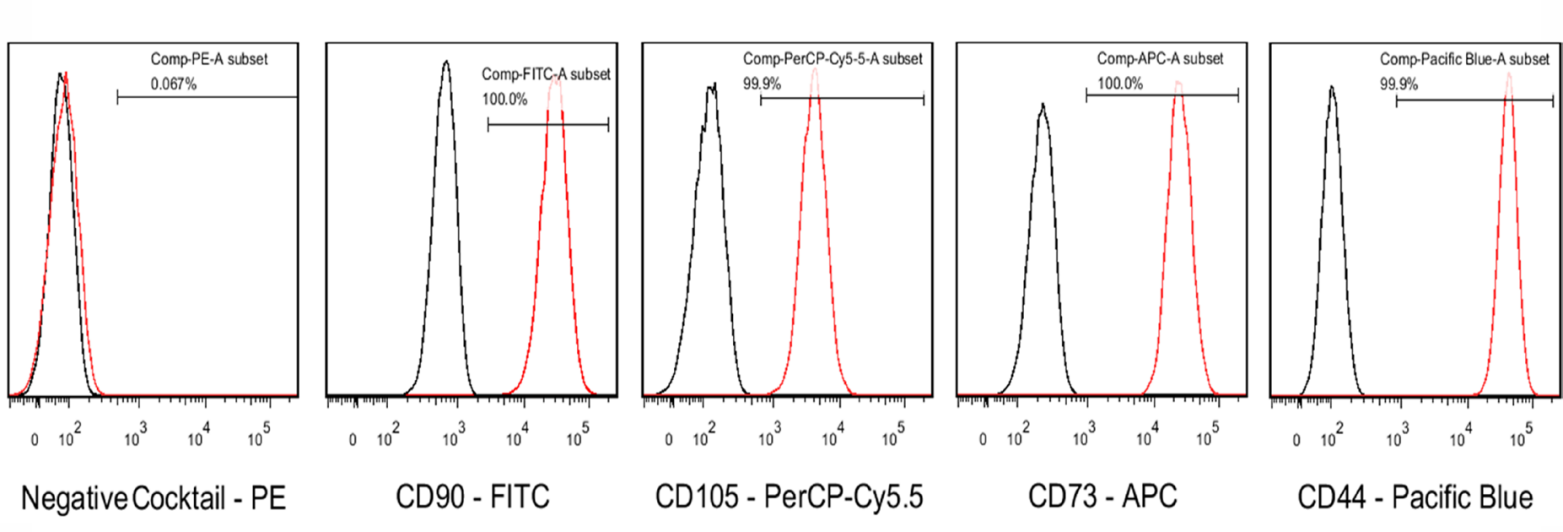
The expression of PDMSC-specific CD cell surface markers was analyzed by flow cytometry The BD stemflow^TM^ human MSC Analysis kit was used to analyze MSC-specific cell surface markers (CD44, CD73, CD105, and CD90).

**Figure 6:**
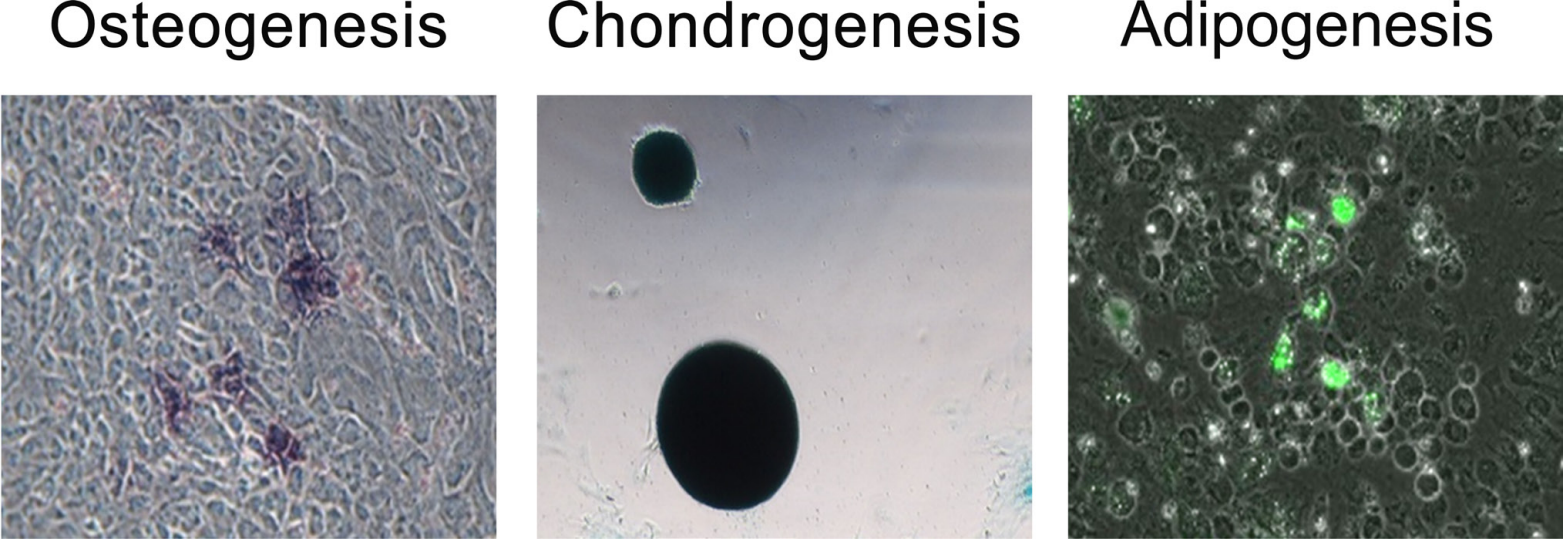
Tri-lineage differentiation analysis was performed to evaluate the differentiation capacity of human PDMSCs (from left to right: osteogenesis, chondrogenesis, and adipogenesis) Alkaline phosphatase (Sigma, B6404), Alcian blue (Sigma, A5268), and the AdipoRed Assay Reagent (Lonza, PT-7009) were used to stain osteocytes, chondrocytes, and adipocytes, respectively.

Flow cytometry analysis was performed using a BD FACSCanto II flow cytometer (Becton Dickinson Company, Franklin Lakes, NJ, USA). PDMSCs were CD44^+^, CD73^+^, CD90^+^, and CD105^+^, and CD34^−^, CD45^−^, CD11b^–^, and HLADR^–^ (Figure 5). The cells were capable of differentiating into osteoblasts, adipocytes, and chondrocytes (Figure 6).

### PDMSCs preparation

PDMSCs were removed from the liquid nitrogen tank and thawed in a 37°C water bath. Once thawed, the cells were mixed with culture media and plated at a density of 3,000 cells/cm^2^ in a T175 flask. Once the cells reached 80% confluence, they were trypsinized for 2 minutes at 37°C and the trypsin neutralized with complete culture media. The cell suspension was centrifuged at 300 RCF for 5 minutes. The supernatant was aspirated and the cell pellet resuspended in normal saline. The final concentration was adjusted to 1 × 10^6^ cells/mL.

### Experimental groups

Animals were randomly divided into five groups: a (i) normothermic control (NC) group treated with vehicle solution (V) (1 mL/kg body weight) 1 day before room temperature exposure (V+NC); (ii) normothermic control group treated with PDMSCs (1 × 10^6^/mL/kg body weight) 1 day before room temperature exposure (PDMSCs+NC); (iii) heat stroke (HS) rats treated with vehicle solution (1 mL/kg body weight) 1 day before heat stress (V+HS); (iv) HS rats treated with PDMSCs (1 × 10^6^/mL/kg body weight) 1 day before heat stress (PDMSCs+HS); and (v) HS rats treated with PDMSCs (1 × 10^6^/mL/kg body weight) immediately after heat stress (PDMSCs[post]+HS). Each group consisted of 10 animals, which was the minimum to achieve statistical significance (*P* < 0.05).

### Measurement of the extent of hypothalamic ischemia

Animals were positioned in a stereotaxic apparatus (Kopf 1406; Grass Instrument Co., Quincy, MA, USA) and probes inserted to measure local CBF and brain temperature. The probes consisted of a 100-μm diameter thermocouple and two 230 μm fibers. Measurements were acquired using OxyLite and OxyFlo instruments (Oxford optronix, Oxford, UK). The OxyLite 2000 is a two-channel device for measuring temperatures, whereas the OxyFlo 2000 is a two-channel laser Doppler perfusion-monitoring instrument for measuring CBF. The probes were implanted into the hypothalamus using the atlas and coordinates of Paxinos and Watson [37].

In separate experiments, the skull was exposed after a midline excision and a burr hole was made in the skull for the insertion of a microdialysis probe (4 mm in length, CMA/2: I.D. 150 mm, O.D. 220 mm; Carnegie Medicine, Stockholm, Sweden). The microdialysis probe was implanted stereotaxically into the hypothalamus. Microdialysates were collected every 10 min in a CMA/140 fraction collector (Carnegie Medicine, Stockholm, Sweden). Aliquots of the microdialysates (5 μL) were injected into a CMA600 Microdialysis Analyzer (Carnegie Medicine) and markers of cellular ischemia including late/pyruvate and glutamate measured [38–40].

### Measurement of core body temperature and MAP in heat stroke rats

Body core temperature was measured by inserting a thermocouple into the rectum of the animal. The MAP was measured by connecting the arterial cannulation (PE50 tube) to a transducer and a four-channel polygraph.

### Measurement of the serum levels of lipid peroxidation indicators and pro-inflammatory cytokines in heat stroke rats

Blood samples were collected 30 min after termination of heat stress and centrifuged at 12,000 rpm. The supernatants were collected and stored at −80°C until use in experiments. Lipid peroxidation was assessed by mixing serum with thiobarbituric acid to form a chromophore (absorbance at 532 nm) and measuring MDA levels using the Esterbauer method [41]. The levels of pro-inflammatory cytokines including TNF-α and IL-1β in serum were measured using double antibody sandwich enzyme-linked immunosorbent assays according to the manufacturer's instructions (R&D Systems, Minneapolis, MN, USA).

### Statistical analyses

The data for multiple independent experiments are expressed as the mean ± standard deviation (S.D.). Survival rates were compared using Kaplan-Meier analysis followed by log-rank tests. One-way analysis of variance followed Student-Newman-Keuls post-hoc tests was performed to analyze differences between multiple groups. A *P* < 0.05 was considered significant.

### Ethics statement

This study was approved by the Ethics Committee for Clinical Research at Chi Mei Medical Center (Tainan, Taiwan) (IRB serial no. 10405–008). All of the experimental protocols were approved by the Institutional Animal Care and Use Committee of Chi Mei Medical Center (Tainan, Taiwan) (ICAUC Approval No. 104042801). The study followed the Institutional and the National Ministry of Science and Technology guidelines for laboratory animal care.

## Abbreviations

PDMSCs: human placenta-derived mesenchymal stem cells
HS: heat stroke
MAP: mean arterial pressure
HR: heart rate
V: vehicle
NC: normothermic control
CBF: cerebral blood flow
MDA: malondialdehyde
TNF-α: tumor necrosis factor alpha
IL-1β: interleukin-1 β
ICP: intracranial pressure
CPP: cerebral perfusion pressure.
